# Pore elimination mechanisms during 3D printing of metals

**DOI:** 10.1038/s41467-019-10973-9

**Published:** 2019-07-12

**Authors:** S. Mohammad H. Hojjatzadeh, Niranjan D. Parab, Wentao Yan, Qilin Guo, Lianghua Xiong, Cang Zhao, Minglei Qu, Luis I. Escano, Xianghui Xiao, Kamel Fezzaa, Wes Everhart, Tao Sun, Lianyi Chen

**Affiliations:** 1 Department of Mechanical and Aerospace Engineering, Missouri University of Science and Technology, Rolla, MO 65409 USA; 2 Department of Materials Science and Engineering, Missouri University of Science and Technology, Rolla, MO 65409 USA; 3 X-ray Science Division, Advanced Photon Source, Argonne National Laboratory, Lemont, IL 60439 USA; 4 Department of Mechanical Engineering, National University of Singapore, Singapore, 117575 Singapore; 5 Department of Energy’s Kansas City National Security Campus Managed by Honeywell FM&T, Kansas City, MO 64147 USA

**Keywords:** Metals and alloys, Design, synthesis and processing

## Abstract

Laser powder bed fusion (LPBF) is a 3D printing technology that can print metal parts with complex geometries without the design constraints of traditional manufacturing routes. However, the parts printed by LPBF normally contain many more pores than those made by conventional methods, which severely deteriorates their properties. Here, by combining in-situ high-speed high-resolution synchrotron x-ray imaging experiments and multi-physics modeling, we unveil the dynamics and mechanisms of pore motion and elimination in the LPBF process. We find that the high thermocapillary force, induced by the high temperature gradient in the laser interaction region, can rapidly eliminate pores from the melt pool during the LPBF process. The thermocapillary force driven pore elimination mechanism revealed here may guide the development of 3D printing approaches to achieve pore-free 3D printing of metals.

## Abstract (web summary)

3D printing pore-free complex metal parts remains a challenge. Here, the authors combine in-situ imaging and simulations to show thermocapillary force can eliminate pores from the melt pool during a laser powder bed fusion process.

## Introduction

Laser powder bed fusion (LPBF) is a 3D printing technology (also known as additive manufacturing) that can print metal parts with complex geometries directly from digital models without the design constraints of traditional manufacturing routes, which has the potential to revolutionize biomedical, aerospace, and defense industries^1–3^. However, the parts printed by the LPBF normally contain many more pores than those made by conventional methods^4^, which severely hinders their applications, because pore is one of the most detrimental defects that cause failure of parts^5^. Many mechanisms can cause pores to form in the melt pool during the printing process (e.g., pore transfer from feedstock powder^6^, instability of depression zone during printing process^7^, vaporization of volatile elements^8^, gas precipitation^9^). Those pores in the melt pool cannot be effectively eliminated by buoyant force^9^, a commonly used mechanism that eliminates pores from liquid^10^, because the high drag force, that is induced by the strong melt flow in the LPBF process, traps the pores within the melt pool^11^. Thus, pores have been ubiquitously observed in as-printed parts^4^. It is very challenging to completely eliminate pores in the printed parts by post processing. For example, the hot isostatic pressing (HIP) cannot close the surface pores^5^; and the gas pores closed by HIP can reopen and grow during subsequent heat treatment^12^.

Therefore, it is critical to uncover the dynamics and mechanisms of pore evolution and elimination in the melt pool during the LPBF process and identify mechanisms for eliminating pores during the printing process, in order to obtain as-printed parts with very low or zero porosity. However, because of the small sizes and high velocity of the pores, as well as the opaque nature of metals, it has been very challenging to probe the motion of these micro-pores in-situ and in real time. Earlier works, involving the use of X-ray imaging to visualize pore motion in a laser melt pool, achieved some success^11,13,14^, but the resolutions afforded by a lab source, or a mid-energy synchrotron facility, are not sufficient to capture some of the faster motions of those micro-pores.

Here, we reveal the highly dynamic and complex motions of micro-pores in the melt pool during LPBF process by using the high-speed hard X-ray imaging technique, with high resolutions (100 ps temporal resolution and ~2 µm spatial resolution). With complementary multi-physics modeling, we find that the pore moving behavior is governed by the competition of the temperature gradient induced thermocapillary force and the melt flow induced drag force. We identify that the high thermocapillary force induced by the high temperature gradient in the laser interaction region can overcome the drag force induced by melt flow to rapidly eliminate pores from the melt pool during LPBF process. The thermocapillary force driven pore elimination mechanism revealed here could be used to design 3D printing approaches to achieve pore-free 3D printing of metals.

## Results

### In-situ characterization of pore dynamics during LPBF

**Fig. 1:**
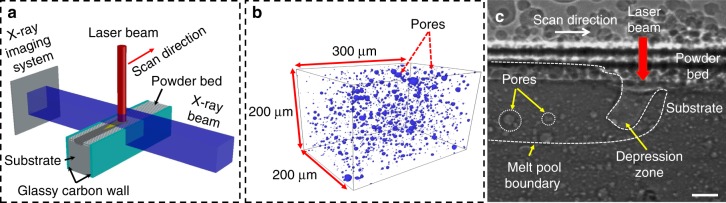
In-situ characterization of pore dynamics during LPBF process. **a** Schematic illustration of the in-situ high-speed X-ray imaging experiment. **b** Representative cuboid (300 µm × 200 µm × 200 µm) reconstructed from X-ray computed tomography data showing the size and distribution of pores inside an additively manufactured AlSi10Mg plate. **c** Representative single-pulse X-ray image revealing micro-pores as well as the melt pool and depression zone beneath the surface of the powder bed (laser power of 360 W, scan speed of 1 m s^−1^ and laser beam diameter (*D*4*σ*) of 100 μm). The boundaries of the melt pool and the depression zone are indicated by a white dashed line, and the position of the laser is indicated by a red arrow. The scale bar in **c** is 50 μm

The in-situ high-speed X-ray imaging experiment to capture the dynamics of pore motion and elimination during LPBF is schematically shown in Fig. 1a. The in-situ X-ray imaging experiment setup consists of a powder bed system (a 100 µm layer of powder on a substrate sandwiched between two glassy carbon plates), a selective laser melting system (to scan the powder bed and create a melt pool), and a high-speed X-ray imaging system (to capture the dynamics of the LPBF process)^15–17^. In order to probe pore motion in every location in the melt pool, AlSi10Mg plate samples, with uniformly dispersed pores (diameters of 10–60 µm), were built by the LPBF as the substrates, as shown in Fig. 1b. Single-pulse (100 ps pulse width) X-ray imaging was conducted with a recording rate of 135,776 frames per second (see “Methods” section for details). A representative single-pulse X-ray image is depicted in Fig. 1c, and a representative X-ray movie is presented in Supplementary Movie 1. Absorption and phase contrast in the X-ray image, generated by different features, permit an easy identification of the micro-pores, melt pool boundary, and vapor depression zone, as indicated in Fig. 1c. Meanwhile, the high spatial and temporal resolutions afforded by the 3rd generation high-energy synchrotron facility and the state-of-the-art beamline instruments have enabled the quantification of the pore moving trajectories at different locations in the melt pool (Supplementary Movie 1). Experiments on AlSi10Mg bare substrates (i.e., without powders on top) were also conducted to investigate and determine the pore dynamics relevant to the non-powder type 3D printing processes, (e.g., laser foil printing)^18^. One example is shown in Supplementary Movie 2. We conducted in-situ experiments under various processing conditions (i.e., various laser powers, scan speeds, and layer thicknesses), and observed similar pore motion behaviors. Here, we will focus on the results obtained under laser power of 360 W, laser scan speed of 1 m s^−1^, and layer thickness of 100 µm to demonstrate the dynamics and mechanisms of pore motion and elimination during the LPBF process.

### Dynamics of pore motions within melt pool

**Fig. 2:**
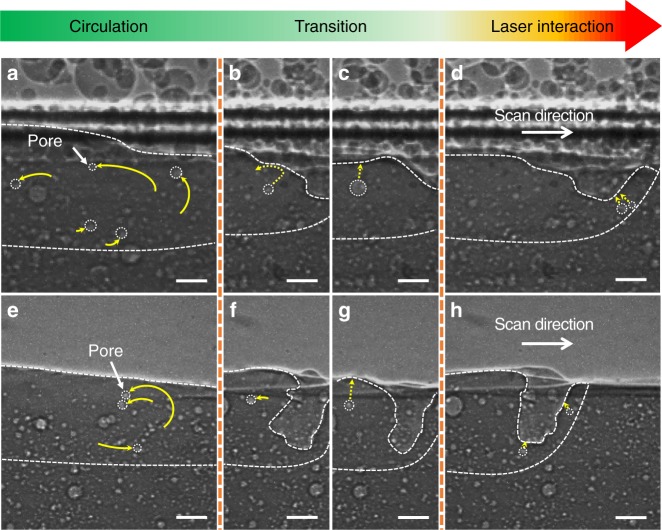
Dynamic pore motions within melt pool. **a**–**d** X-ray images showing pore dynamics during the LPBF process. The thickness of powder layer is 100 μm. **e**–**h** X-ray images showing pore dynamics during laser melting of a bare substrate. Dotted arrows indicate the future trajectories of the pores, while solid arrows mark the history of pore trajectories. Pores follow circular patterns at the circulation domain (**a**, **e**), while pores in the laser interaction domain move toward depression zone and escape from the melt pool (**d**, **h**). In the transition domain (**b**, **c**, **f** and **g**), pores exhibit irregular moving behavior, sometimes moving toward the melt pool surface and escaping (**c**, **g**), and sometimes circulating in the melt pool (**b**, **f**). The laser beam diameter (*D*4*σ*) is 100 μm, the laser power is 360 W, and the scan speed is 1 m s^−1^. All scale bars are 50 μm

The movements of individual pores in the melt pool were carefully traced, and it was found that the pores in different regions of the melt pool exhibited different moving patterns (Fig. 2a–d and Supplementary Movie 1). In the region near the laser beam, the pores moved toward depression zone and escaped from the melt pool, hereafter referred to as the laser interaction domain (Fig. 2d). In the region at a certain distance away from the laser interaction domain, the pores circulate within the melt pool, hereafter referred to as the circulation domain (Fig. 2a). Between these two regions, the pores move in irregular patterns, i.e., sometimes moving toward the surface of the melt pool and escaping (Fig. 2c), while sometimes circulating in the melt pool (Fig. 2b), hereafter referred to as the transition domain. Similar pore moving behaviors were observed during the laser melting of AlSi10Mg bare substrates (Fig. 2e–h and Supplementary Movie 2), as well as under other processing conditions (another example is shown in Supplementary Fig. 1).

### Driving forces for pore motion and elimination

**Fig. 3:**
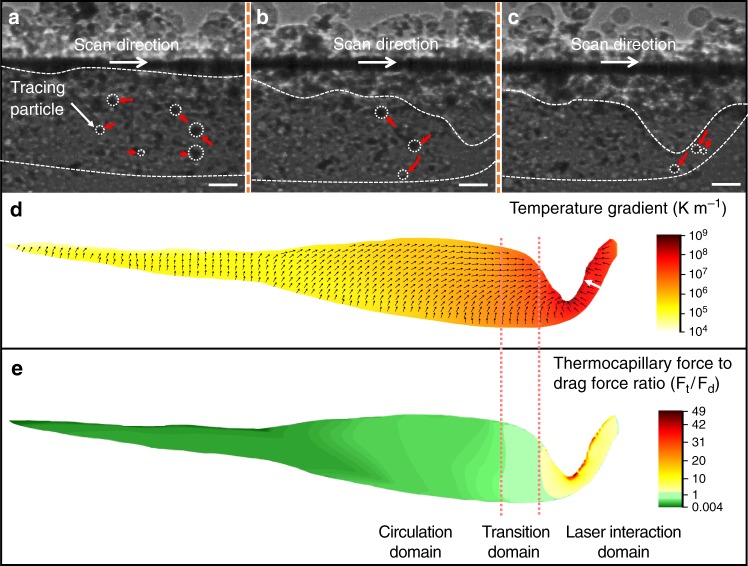
Driving forces for pore motion and elimination. **a**–**c** X-ray images showing trajectories (indicated by red arrows) of the tracing particles (tungsten microparticles, marked by white dotted circles), which indicate the melt flow at the circulation domain (**a**) transition domain (**b**) and laser interaction domain (**c**) inside the melt pool during the LPBF process. **d** Temperature gradient inside the melt pool during LPBF process, obtained by multi-physics modeling with laser processing parameters the same as the in-situ experiments (see “Methods” section). The magnitude and direction of temperature gradient are indicated by color and black arrows, respectively. The white arrow indicates the temperature gradient increases from the solid-liquid interface (melting front) to the depression zone front wall (Fig. 5). **e** Ratio of thermocapillary force (F_t_) to drag force (F_d_) for a pore with a diameter of 10 µm. In **a**–**c**, the laser power is 360 W, the scan speed is 1 m s^−1^ and the thickness of powder layer is 100 μm. Scale bars in **a**–**c** are 50 μm

We have initially tried to explain the pore moving dynamics observed using the mechanisms reported in the literature: pore motion driven by buoyant force and melt flow induced drag force^9,11^. The buoyant force was calculated directly based on the actual pore size measured from the X-ray images. The estimation of melt flow induced drag force, on the other hand, needs the flow velocity as input, which is challenging to measure experimentally^1^. Here, a particle tracing experiment was designed and carried out to characterize the melt flow velocity within the melt pool during LPBF using high-speed x-ray imaging. Specifically, tungsten microparticles (1 wt.%, diameter ≤ 10 μm) were embedded in AlSi10Mg powders as tracing markers, and the melt flow behavior was quantified based on the velocity of tungsten microparticles in different domains, as shown in Fig. 3a–c. In the circulation domain, the melt circulates with an average velocity of 0.6 ± 0.2 m s^−1^ (mean ± standard deviation (s.d)) (Fig. 3a). In the laser interaction domain, the melt flows downward, along the front wall of the vapor depression zone with an average velocity of 1.9 ± 0.6 m s^−1^ (Fig. 3c). In the transition domain, the melt flow pattern is more complex due to the interplay of circulation and backward flow, and exhibits an average velocity of 1.45 ± 0.5 m s^−1^ (Fig. 3b).

**Fig. 4:**
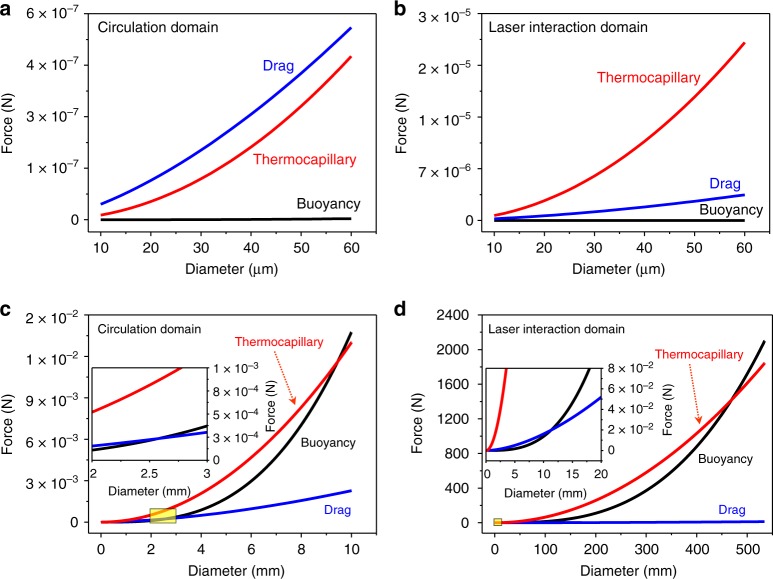
Force analysis. **a**, **b** Comparison of drag force, buoyant force and thermocapillary force at the circulation domain (**a**) and the laser interaction domain (**b**) for the pore size range studied in this work. **c**, **d** Forces as a function of pore diameter. The critical pore size for buoyant force to overcome drag force is 2.5 mm at the circulation domain (**c**) and 11 mm at the laser interaction domain (**d**), which are even larger than the size of a typical melt pool in the LPBF process. The critical pore size for buoyant force to be larger than the thermocapillary force is even larger, 9.5 mm at the circulation domain (**c**) and 470 mm at the laser interaction domain (**d**). The inset (in **c** and **d**) corresponds to the area marked by the yellow rectangle

Based on the measured melt flow velocity, the drag force was calculated. The results indicate that the drag force is orders of magnitude higher than the buoyant force for the pore size range studied here (Fig. 4a, b). Thus, it is expected that pores in the melt pool will move with the melt flow, and have very limited opportunity to float up and escape. This can satisfactorily explain pore moving dynamics in the circulation domain. However, in the laser interaction domain, the pores move approximately perpendicular to the melt flow direction and manage to rapidly escape out from the melt pool, with a velocity of up to over 2 m s^−1^, even though the melt flow velocity is the highest in this domain among all locations in the melt pool. This is in contrary to pore motion behavior predicted based on buoyant force and melt flow induced drag force.

**Fig. 5:**
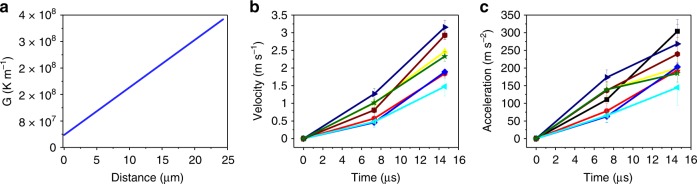
Increase of acceleration as pores move toward depression zone wall. **a** Variation of temperature gradient, *G*, from the melting front (solid-liquid interface during melting) to the depression zone wall (indicated by the white arrow in Fig. 3d). The temperature gradient increases towards the depression zone wall. **b** Velocity, as a function of time, as pores move toward depression zone wall. **c** Acceleration, as a function of time, as pores move toward depression zone wall. The acceleration increases as pores move toward the depression zone wall. Error bars represent standard deviation (s.d.); The data in **b** and **c** are collected during laser melting of an AlSi10Mg bare plate (laser power of 310 W, scan speed of 1 m s^−1^)

After carefully analyzing the pore moving direction and the temperature gradient, and inspired by the thermocapillary force driven liquid droplet movement in immiscible alloys^19,20^, we hypothesize that the thermocapillary force induced by the high temperature gradient in the laser interaction domain is the driving force for the unexpected pore elimination. For the material we studied, as well as most metals and alloys, the temperature coefficient of surface tension is negative. The thermocapillary force drives the melt around the pore flows from hot region to cold region. As a result, the pore moves from cold region to hot region. In order to calculate the thermocapillary force, the temperature gradient in the melt pool was simulated by a multi-physics model (see Supplementary Fig. 2, Supplementary Tables 1, 2 and Supplementary Movie 3)^21–23^. In the laser interaction domain, the temperature gradient exhibits an average value of 6.5 × 10^7^ K m^−1^ with a direction approximately normal to the melt pool surface (indicated by small black arrows in Fig. 3d). Such high temperature gradient results in a thermocapillary force that is at least three times higher than the melt flow induced drag force (see Fig. 4b for details) in the laser interaction domain. Therefore, the pores in the laser interaction domain move along the temperature gradient and escape from the melt pool. Detailed analysis reveals that the temperature gradient increases from the melting front to the vapor depression front wall, as indicated by a white arrow in Fig. 3d. This implies that the acceleration of a pore will increase when it moves toward the depression zone. In the experiment, this predicted acceleration increase was indeed observed, which further confirms the thermocapillary force driven pore elimination mechanism proposed here (Fig. 5).

To study the effect of thermocapillary force on pore dynamics in different locations of the entire melt pool, we developed a force map based on the ratio of thermocapillary force to drag force (F_t_/F_d_) for a 10 μm-dimeter pore, using the local temperature gradient and the average velocity of the melt flow (1.1 ± 0.5 m s^−1^). The buoyant force is neglected because it is orders of magnitude smaller than the thermocapillary force and the drag force for the pore size range studied here. As shown in Fig. 3e, the F_t_/F_d_ ratio varies significantly at different locations in the melt pool due to significant variations in the temperature gradient. F_t_/F_d_ ranges from the highest value of ~47 in the laser interaction domain to ~0.004 near the tail of the melt pool. Thus, the thermocapillary force is the dominating force in the laser interaction domain, which drives the pores to move in the direction of the temperature gradient, while the drag force controls pore motion in the circulation domain. In the transition domain, F_t_ and F_d_ are very close, which results in the irregular and ambivalent pore moving behavior.

### Mechanisms of pore dynamics and elimination

**Fig. 6:**
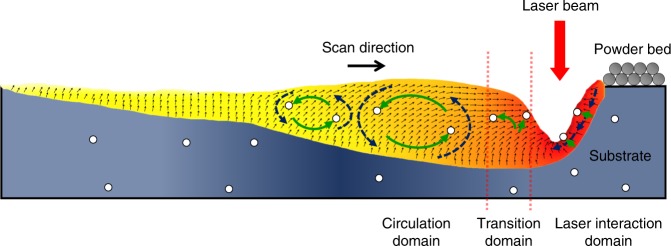
Mechanisms of pore dynamics and elimination. Schematic illustration showing the dynamics of pore motion and mechanisms of pore elimination during the LPBF process

The dynamics and mechanisms of pore motion and elimination in the melt pool during the LPBF process is schematically summarized in Fig. 6. The pore moving behavior is governed by competition of the temperature gradient induced thermocapillary force and the melt flow induced drag force. The buoyant force will play a more important role when the size of the pore becomes larger. However, our estimation shows that, for the buoyant force to become dominant under normally used LPBF condition, the sizes of the pores need to reach millimeters, even larger than the size of a typical melt pool in the LPBF process (Fig. 4c, d). Thus, the main driving force for pore elimination during the LPBF process is the thermocapillary force, instead of the commonly thought buoyant force.

### Eliminating pores using thermocapillary force

**Fig. 7:**
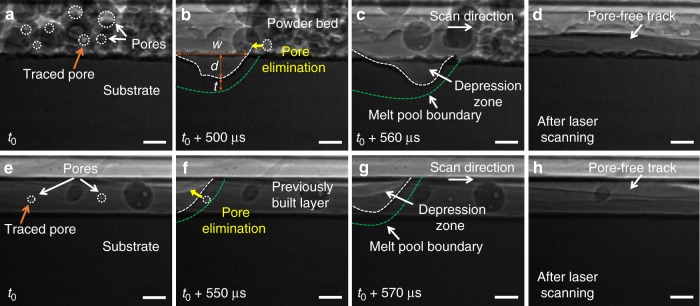
Eliminating pores using thermocapillary force. **a**–**d** Dynamic X-ray images showing elimination of pores in feedstock powders. The thickness of powder layer is 100 μm. In **b** *w* and *d* indicate the depression zone width and depth, respectively, and *t* represents the thickness of the liquid layer around the depression zone. **e**–**h** Dynamic X-ray images showing elimination of pores in the previously built layer. Traced pores are indicated by orange arrows. Pore moving trajectories are indicated by yellow arrows. The melt pool and depression zone boundaries are outlined by green and white dashed-lines, respectively. The powder and substrate are Ti6Al4V. The laser beam diameter (*D*4*σ*), laser power, and scan speed are 75 μm, 210 W, and 0.6 m s^−1^, respectively. All scale bars are 50 μm

The thermocapillary force driven pore elimination can serve as an effective approach to eliminating pores during the LPBF process. Here, two examples are presented as a proof of concept. First, we show that the pores in the feedstock powders can be eliminated by thermocapillary force under proper laser processing conditions to achieve a pore-free track, as shown in Fig. 7a–d and Supplementary Movie 4. Second, we demonstrate that the pores in the previously built layer will be eliminated by thermocapillary force by laser rescanning with proper laser scan parameters, as shown in Fig. 7e–h and Supplementary Movie 5. We have performed experiments on both AlSi10Mg and Ti6Al4V alloys. We achieved pore elimination by thermocapillary force in both alloys, which indicates that the thermocapillary force driven pore elimination mechanism is not limited to a specific alloy system.

The proper laser processing parameters are determined based on the following two general guidelines. First, the temperature gradient in the laser interaction domain is high enough to overcome the melt flow induced drag force. The temperature gradient around the laser interaction domain can be estimated by the difference between the boiling temperature (*T*_b_) and melting temperature (*T*_m_) of the material over the thickness (*t*, as indicated in Fig. 7b) of the liquid layer around the depression zone, (*T*_b_−*T*_m_)/*t*. For a given material, a smaller *t* indicates a higher temperature gradient. Second, the area of the high temperature gradient region should be reasonably large to have a good opportunity to encounter pores. This means a larger laser interaction domain, which can be estimated by the width (*w*) over depth (*d*) ratio of the depression zone, *w*/*d*, as indicated in Fig. 7b. However, the depression zone depth, *d*, cannot be too small. When *d* is too small, the melt pool depth is too shallow, which may cause lack of fusion. The proper laser processing parameters to eliminate the pores for the examples shown in Fig. 7 are obtained based on the above two general guidelines using in-situ x-ray imaging.

In summary, we find a mechanism for effectively eliminating pores in 3D printing of metals by synergistically combining sophisticated in-situ experiments and multi-physics modeling. We expect that the thermocapillary force driven pore elimination mechanism revealed here could open avenues for developing approaches to achieve pore-free 3D printing to unleash the full potential of 3D printing technologies. The thermocapillary force driven pore elimination mechanism also has implications for a broad range of research and engineering fields where pore evolution is important and a temperature gradient exists, such as laser polishing^24^, laser cladding^25^, welding^26^, melt spinning^27^, reactions in nuclear reactors^28^, and chemical reactors^29^.

## Methods

### Materials and sample preparation

AlSi10Mg rectangular bars (40 mm long × 8 mm wide × 8 mm thick), containing uniformly distributed pores with diameters ranging from 10 to 60 μm (Fig. 1b) for pore dynamics study, were fabricated with a powder bed fusion additive manufacturing machine, Renishaw AM250 (Renishaw, UK). The substrate used in Fig. 7 for pore elimination study is commercial Ti6Al4V sheet. The substrates for X-ray imaging experiments (40 mm long × 3 mm wide × 0.5–1.1 mm thick) were cut using wire electrical discharge machining (EDM) and then polished down to 0.3–1 mm thick using silicon carbide sandpaper. The AlSi10Mg powders (15–45 μm, LPW Technology, Ltd, UK) and Ti6Al4V powders (53–106 μm, PRAXAIR, USA) were spread on the substrates to form the powder bed for in-situ high-speed x-ray imaging experiments.

### High-speed X-ray imaging

High-speed high-resolution hard X-ray imaging was performed at beamline 32-ID-B of the Advanced Photon Source at Argonne National Laboratory to monitor the pore dynamics inside a melt pool in real time. The schematic of the synchrotron x-ray experiment is shown in Fig. 1a. The samples studied in the experiments were miniature powder beds. A typical sample is composed of a piece of substrate sandwiched between two glassy carbon plates, and a 100 μm-thick layer of powder. A continuous-wave (CW) ytterbium fiber laser (IPG YLR-500-AC, IPG Photonics, Oxford, USA, wavelength of 1070 nm, maximum output power of 520 W) and a galvo scanner (Intelli*SCAN*_de_ 30, SCANLAB GmbH., Germany) were integrated to perform single track laser melting on both powder bed and bare substrate under various laser powers (from 100–520 W) and scan speeds (0.2–1.5 m s^−1^).

The X-ray beam used in the experiments was an undulator-generated pseudo pink beam with the 1st harmonic energy at 24 keV. The X-ray beam penetrated through metal samples. The transmitted beam was captured by a detection system downstream where the X-ray signal was converted into a visible light image using a single crystalline scintillator (Lu_3_Al_5_O_12_:Ce, 100 µm thickness), and then recorded by a high-speed camera (Photron FastCam SA-Z, Japan) with a 10× objective lens. The nominal spatial resolution is 2 μm/pixel. In these experiments, the images were recorded with a frame rate of 135.776 kHz. The images were processed using ImageJ^30^. All experiments were conducted in a custom-built experimental chamber filled with argon gas (1 atm pressure).

### X-ray micro computed tomography (µ-CT)

X-ray μ-CT was used to characterize the pore size and distribution in the AlSi10Mg substrate three-dimensionally (Fig. 1b). The experiments were conducted at beamline 2-BM-A of the Advanced Photon Source at Argonne National Laboratory. A pink X-ray beam, with the energy centered at 25 keV penetrated through the sample and was converted to a visible light signal using a single crystalline scintillator (Lu_3_Al_5_O_12_:Ce, 20 µm thickness). Then, the visible light signal was captured using a CMOS camera (pco.edge camera, PCO AG, Kelheim, Germany) with a 10× objective lens. The effective pixel resolution is 650 nm. 1500 projection images were recorded over the 180° rotation of the sample, with an exposure time of 0.1 s for each image. The rotation speed of the stage was maintained at 1° per second. The through-the-thickness slices were reconstructed from the projections using an in-house software (Tomopy^31^). These slices were then stitched together using image processing software (Avizolite 9.4, FEI visualization Sciences Group) for the isosurface rendering.

### Characterization of melt flow within melt pool

The melt flow within the melt pool during the LPBF process was characterized using tracing particles. Tungsten microparticles (diameters of ≤10 μm) were embedded in AlSi10Mg powders by ball milling (planetary ball mill, PQ-N04, Across International, LLC.). The velocities of the tungsten microparticles at different locations in the melt pool during the LPBF process were measured using high-speed X-ray imaging (see Supplementary Note 1 on Melt flow analysis with micro-tracing particle). Then, the melt flow velocities in the three domains were estimated. Due to the large variation of melt flow velocities within the circulation domain, two sub-regions in the circulation domain were identified and calculated separately: the melt pool close to the circulation-transition domain boundary and the melt pool tail. Twenty particles’ velocities were measured to calculate the average velocity in each melt flow region.

The average melt flow velocities at the laser interaction domain and the transition domain are 1.9 ± 0.6 m s^−1^ (mean ± standard deviation (s.d.)) and 1.45 ± 0.5 m s^−1^, respectively. Within the circulation domain, the velocity of the melt flow average varies from 0.75 ± 0.2 m s^−1^, at the region near the circulation-transition domain boundary, to 0.4 ± 0.1 m s^−1^, at the melt pool tail. For simplicity, the melt flow velocity within the circulation domain was averaged as 0.6 ± 0.2 m s^−1^, and this value was used to calculate the drag force on the pores in the circulation domain (Fig. 4a, c). To estimate the drag force for developing the force map in Fig. 3e, the average melt flow velocity of the entire melt pool was used (1.1 ± 0.5 m s^−1^, average of 1.9 ± 0.6 m s^−1^, 1.45 ± 0.5 m s^−1^, 0.75 ± 0.2 m s^−1^, and 0.4 ± 0.1 m s^−1^).

### Multi-physics modeling

The temperature in the melt pool was simulated by multi-physics modeling with the laser parameters used in the experiments. The model was calibrated by experimental data (length and depth of melt pool and depth of depression zone). The initial powder bed packing configuration is generated using the Discrete Element Method, where the input powder size distribution follows the experimental measurements and the simulated packing density agrees with experimental measurement. The powder bed geometry is then implemented into a thermal-fluid flow model to simulate the multi-physics process of heat transfer, phase transformation, and molten fluid flow. The fully coupled governing equations, including continuity, momentum, and energy conservation equations, are computed using the Finite Volume Method, while the free surfaces are tracked using the Volume of Fluid method. Flow is assumed to be incompressible, laminar, and Newtonian; laser energy absorption, thermal conduction, surface radiation and convection, and latent heats of melting and vaporization are incorporated for energy conservation. Major driving forces of the molten pool flow are implemented, including recoil pressure, surface tension, Marangoni effect, viscosity, buoyancy, and gravity. The thermophysical properties of AlSi10Mg used for simulation are given in Supplementary Tables 1 and 2. Further details about these models can be found in the reference^21–23^.

### Force calculation

The buoyant force (**F**_b_), melt flow induced drag force (**F**_d_), temperature gradient induced thermocapillary force (**F**_t_) were calculated using the equations reported in the literature. The equation for calculating each force is discussed below (bold characters denote vector quantities):

The buoyant force (**F**_b_) is calculated by equation^32^:

1 $${\bf{F}}_{\mathrm{b}} = \frac{4}{3}{\mathrm{\pi }}\,{\it{r}}_{\mathrm{p}}^3\,{\it{\rho }}_{\mathrm{f}}\,{\bf{g}}$$

where *r*_p_ is the pore radius, *ρ*_f_ is the melt density and *g* is the gravitational acceleration (*g* *=* 9.8 m s^−2^).

The drag force (**F**_d_) is induced by melt flow, which consists of a form drag and friction drag. When Reynold number is higher than unity, the drag force is calculated by the following equation^33^:

2 $${\bf{F}}_{\mathrm{d}} = - \frac{1}{2}C_{\mathrm{D}}\,\rho _{\mathrm{f}}\,r_{\mathrm{p}}^2\left( {{\bf{U}}_{\mathrm{p}} - {\bf{U}}_{\mathrm{f}}} \right)\left| {{\bf{U}}_{\mathrm{p}} - {\bf{U}}_{\mathrm{f}}} \right|$$

where *r*_p_ is the pore radius, *ρ*_f_ is melt density, **U**_p_ is the pore velocity vector, **U**_f_ is the melt flow velocity vector, and *C*_D_ is the drag coefficient (dimensionless) which depends on the melt flow regime and molten metal properties and is approximated by Schiller and Naumann equation:

3 $$C_{\mathrm{D}} = \frac{{{\mathrm{24}}}}{{{\mathrm{Re}}}}\left( {1 + {\mathrm{0}}{\mathrm{.15Re}}^{0.687}} \right)$$

where Re is Reynold number and is given by:

4 $${\mathrm{Re}} = \frac{{2\,r_{\mathrm{p}}\,\rho _{\mathrm{f}}\left| {{\mathbf{U}}_{\mathrm{p}} - {\mathbf{U}}_{\mathrm{f}}} \right|}}{{\it{\mu }}}$$

where *r*_p_ is pore radius, **U**_p_ is the pore velocity vector, **U**_f_ is the melt flow velocity vector*, ρ*_f_ and *μ* are melt density and melt dynamic viscosity, respectively. We calculated the drag force exerted on the pore with zero velocity (|**U**_p_| = 0) to construct the force map.

Thermocapillary force (**F**_t_) is induced by the temperature gradient around the pore. Thermocapillary force is calculated by the following equation^34^:

5 $${\bf{F}}_{\mathrm{t}} = {\mathrm{4}}\,\pi \,{\it{r}}_{\mathrm{p}}^2\,\frac{{\partial T}}{{\partial r}}\frac{{\partial {\mathrm{\sigma }}}}{{\partial T}}$$

where $\frac{{\partial T}}{{\partial r}}$ and $\frac{{\partial \sigma }}{{\partial T}}$ are the temperature gradient at the location of the pore and the temperature coefficient of surface tension, respectively. Due to lack of data on the temperature coefficient of surface tension of AlSi10Mg alloy, the temperature coefficient of surface tension of Al_88_Si_12_ alloy $\left( {\frac{{\partial \sigma }}{{\partial T}} = - 0.31 \times 10^{ - 4}{\mathrm{N}}\,{\mathrm{m}}^{ - 1}{\mathrm{K}}^{ - 1}} \right)$ was used^35^. The local temperature gradient values, obtained from the simulation results, were used to calculate the local thermocapillary force for developing the force map in Fig. 3e. The average temperature gradient of 6.5 × 10^7^ K m^−1^ at the laser interaction domain and 1 × 10^6^ K m^−1^ at the circulation domain were used to plot the force-pore diameter graph (Fig. 4).

## Supplementary information

Supplementary information

Supplementary Movie 1

Supplementary Movie 2

Supplementary Movie 3

Supplementary Movie 4

Supplementary Movie 5

Description of Additional Supplementary Files

## Data Availability

The data supporting the findings of this work is available in the main text or supplementary materials. Raw data are available from the corresponding authors on reasonable request.
