# Publisher Correction: Pore elimination mechanisms during 3D printing of metals

**DOI:** 10.1038/s41467-019-12432-x

**Published:** 2019-09-30

**Authors:** S. Mohammad H. Hojjatzadeh, Niranjan D. Parab, Wentao Yan, Qilin Guo, Lianghua Xiong, Cang Zhao, Minglei Qu, Luis I. Escano, Xianghui Xiao, Kamel Fezzaa, Wes Everhart, Tao Sun, Lianyi Chen

**Affiliations:** 1 Department of Mechanical and Aerospace Engineering, Missouri University of Science and Technology, Rolla, MO 65409 USA; 2 Department of Materials Science and Engineering, Missouri University of Science and Technology, Rolla, MO 65409 USA; 3 X-ray Science Division, Advanced Photon Source, Argonne National Laboratory, Lemont, IL 60439 USA; 4 Department of Mechanical Engineering, National University of Singapore, Singapore, 117575 Singapore; 5 Department of Energy’s Kansas City National Security Campus Managed by Honeywell FM&T, Kansas City, MO 64147 USA

**Keywords:** Metals and alloys, Design, synthesis and processing

Correction to: *Nature Communications* 10.1038/s41467-019-10973-9, published online 12 July 2019.

The original version of this Article contained an error in Fig. 4. The *x*-axis labels in Fig. 4a, b were incorrectly labelled ‘Diameter (mm)’, rather than the correct ‘Diameter (µm)’. This has been corrected in both the PDF and HTML versions of the Article.

