# Supplementary material for: Pore elimination mechanisms during 3D printing of metals
Source: Nat Commun. 2019 Jul 12;10:3088. doi: 10.1038/s41467-019-10973-9 (PMC6625989; doi:10.1038/s41467-019-10973-9)
Supplement: Supplementary file 1 — Supplementary information [file 41467_2019_10973_MOESM1_ESM.pdf]

Supplementary Information

Pore elimination mechanisms during 3D printing of metals

S. Mohammad H. Hojjatzadeh et al.

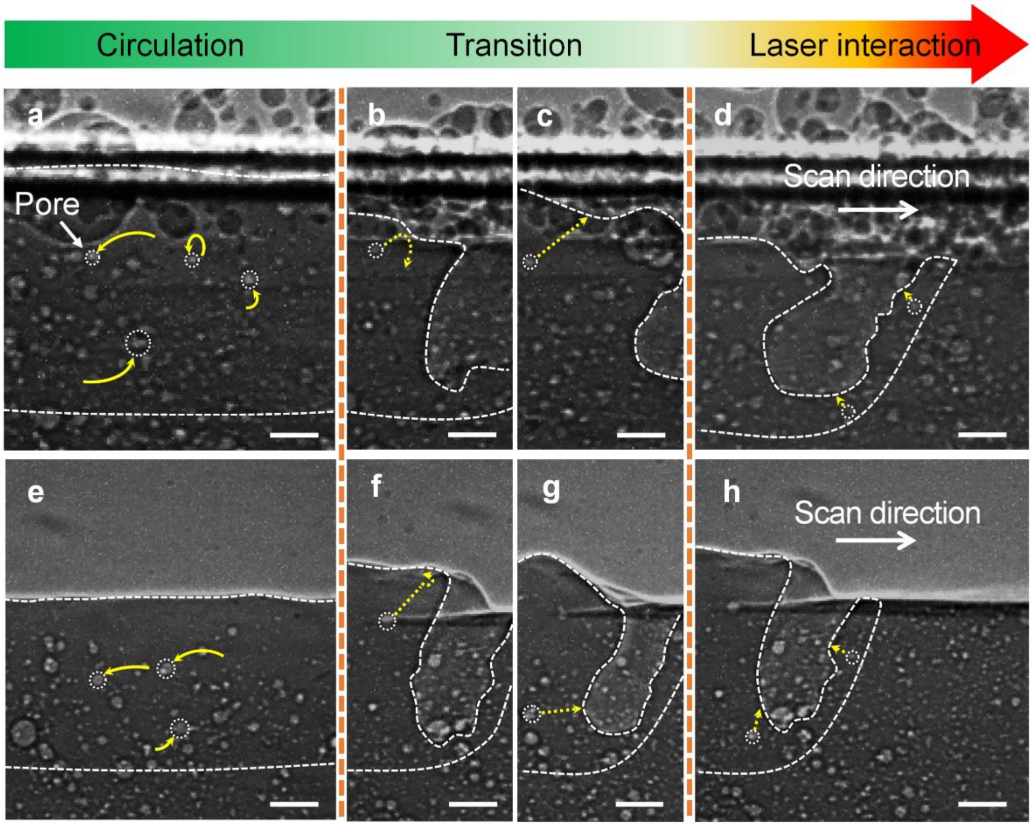

Supplementary Fig. 1. Pore dynamics during laser melting under laser power of 420 W and scan speed of 1 m s^{-1}. **a-d** X-ray images showing pore dynamics during LPBF process. **e-h** X-ray images showing pore dynamics during laser melting of a bare substrate. Dotted arrows indicate the future trajectories of the pores, while solid arrows show the history of pore trajectories. The powder and substrate are AlSi10Mg. In **a-d**, the powder layer thickness is 100 µm. The laser beam diameter (*D*4*σ*) is 100 µm. All scale bars are 50 µm.

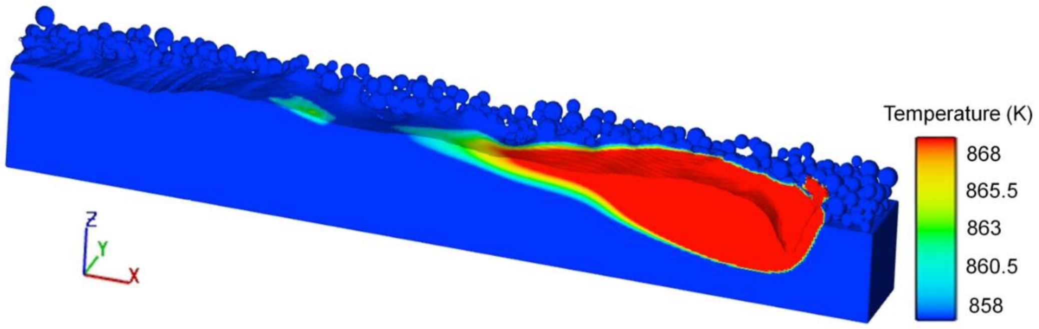

Supplementary Fig. 2. A snapshot showing the multi-physics model. The temperature range was set to highlight the melt pool. The simulation was conducted with the laser parameters used in the in-situ experiments (AlSi10Mg powder and substrate, laser power of 360 W and scan speed of 1 m s^{-1}).

Supplementary Table 1. Thermophysical properties of AlSi10Mg alloy used for simulation[1,2].

Material property	Value	Unit
Solidus temperature, T_s	830.15	K
Liquidus temperature, T_L	870.15	K
Boiling temperature, T_b	2743	K
Density, ρ	2680	kg m^{-3}
Temperature coefficient of surface tension, $\frac{\partial \sigma}{\partial T}$	-0.31×10^{-4}	N m^{-1} K^{-1}
Surface tension at liquidus temperature, σ_L	0.824×10^{-4}	N m^{-1}
Latent heat of fusion, ΔH_f	389×10^{3}	J kg^{-1}
Latent heat of vaporization, ΔH_v	10700×10^{3}	J kg^{-1}
Linear thermal expansion coefficient, α_L	23×10^{-6}	K^{-1}
Convective heat transfer coefficient , h_c	82	W m^{-2} K^{-1}
Radiation emissivity, ε	0.4	
Energy absorptivity, η	0.52	

Supplementary Table 2. Temperature-dependent thermophysical properties of AlSi10Mg alloy used for simulation[1].

Material property	Temperature (K)	Value
Viscosity, v (Pa s)	875	0.0022
	1000	0.00125
	1450	0.0007
	1850	0.00058
	2250	0.00045
	2700	0.0004
Thermal conductivity, k (W m^{-1} K^{-1})	300	160
	400	160
	500	160
	600	160
	700	160
	830	110
	870	90
	1200	100
	1500	110
	1800	115
	2100	120
Specific heat, C_p (J kg^{-1} K^{-1})	300	900
	375	960
	575	1020
	775	1125
	940	1040
	960	1040
	1100	1075
	1562	1075

Supplementary Note 1: Melt flow analysis with micro-tracing particle

To make sure that the tracing particles follow the melt flow velocity, we used tiny tungsten particles (diameter ≤ 10 μm, orders of magnitude smaller than the ones used in previous publications). We have estimated the settling velocity of 10 um tungsten particle in aluminum melt and the time needed for the particle to be accelerated to the velocity close to the melt flow velocity. The analysis results show that the velocity of tracing particles can reflect the velocity of the melt flow: the settling velocity of 10 μm tungsten particle is 3 orders of magnitude slower than the melt flow velocity and it only takes 29 μs for the particle to have a similar speed with the melt flow, as detailed below.

The settling velocity (v_s) of a tungsten particle with diameter of 10 μm in liquid AlSi10Mg is calculated by the Stokes's law[3,4]:

$$v_{\mathrm{s}} = \frac{\rho_{\mathrm{p}} - \rho_{\mathrm{f}}}{\gamma \mu_{\mathrm{f}}} g\, d_{\mathrm{p}}^2 \tag{1}$$

where g is the gravitational acceleration, d_p is the particle diameter, ρ_p is the particle density, ρ_f is the fluid density, μ_f is the fluid dynamic viscosity, and γ is a coefficient which depends on the fluid and particle viscosity: $\gamma = 12[(\mu_f + \frac{3}{2}\mu_p)/(\mu_f + \mu_p)]$. In case of solid particles ($\mu_p = \infty$), γ is equal to 18. Taking g = 9.8 m s^{-2}, $d_p = 10 \times 10^{-6}$ m, ρ_p = 19300 kg m^{-3}, ρ_f = 2680 kg m^{-3}, μ_f = 0.00175 kg (m s)$^{-1}$, the settling velocity of the tungsten particle is calculated to be $v_s = 7.7 \times 10^{-4}$ m s^{-1}, which is three orders of magnitude lower than the average melt flow velocities measured here. Therefore, the settling velocity induced by the buoyancy and gravity on the tungsten particle is negligible.

The time needed to accelerate the tracing particles to the speed similar to the melt flow speed is estimated based on Newton's law using the estimated drag force. We assume a tungsten particle (d_p =10 μm) is accelerated from the top surface at depression-zone front (with an initial speed of 0 m s^{-1}) to the position in front of the depression zone where melt flow speed is 1.9 m s^{-1}. The drag force ($\mathbf{F}_d$) exerted by aluminum melt flow on the tracing particle is calculated by[5]:

$$\mathbf{F}_{\mathrm{d}} = -\frac{1}{2} C_{\mathrm{D}}\, \rho_{\mathrm{f}}\, r_{\mathrm{p}}^2 \left(\mathbf{U}_{\mathrm{p}} - \mathbf{U}_{\mathrm{f}}\right)\left|\mathbf{U}_{\mathrm{p}} - \mathbf{U}_{\mathrm{f}}\right| \tag{2}$$

where r_p is the particle radius, ρ_f is fluid density, $\mathbf{U}_p$ is the particle velocity vector, $\mathbf{U}_f$ is the fluid velocity vector, and C_D is the drag coefficient (dimensionless) which depends on the flow regime and fluid properties and is approximated by Schiller and Naumann equation:

$$C_{\mathrm{D}} = \frac{24}{\mathrm{Re}}(1 + 0.15\mathrm{Re}^{0.687}) \tag{3}$$

where Re is particle Reynold number and is given by:

$$\mathrm{Re} = \frac{2\, r_{\mathrm{p}}\, \rho_{\mathrm{f}} \left|\mathbf{U}_{\mathrm{p}} - \mathbf{U}_{\mathrm{f}}\right|}{\mu} \tag{4}$$

where r_p is the particle radius, $\mathbf{U}_p$ is the particle velocity vector, $\mathbf{U}_f$ is the fluid velocity vector, ρ_f and μ are fluid density and fluid dynamic viscosity, respectively.

Taking $\left|\mathbf{U}_\mathrm{p} - \mathbf{U}_\mathrm{f}\right|$ = 1.9 m s^{-1}, ρ_f = 1740 kg m^{-3} (near T_b = 2743 K), μ = 0.0004 kg $(m.s)^{-1}$ (near T_b = 2743 K), the drag force is calculated to be 3.2 μN. Considering that the drag force will decrease when the speed difference between the tracer and the melt flow becomes smaller, the total acceleration time can be estimated by iteration with small time steps. Assuming the initial tracer speed is zero, the surrounding melt flow speed is 1.9 m s^{-1} and the iteration time step is set to be 10^{-9} s, it is calculated that the tungsten particle with diameter of 10 μm can be accelerated from zero speed to approximately 85% of the surrounding melt flow speed within 29 μs (about 4 frames). Here, the assumption was that the initial speed of the particle was zero and diameter of tungsten particle is 10 μm. However, in most cases the initial speed of tungsten particle is not zero and the particle diameter is smaller than 10 μm. As a result, the particle can reach the similar melt flow speed at a much shorter time.

Supplementary References

1. Wei, P., Wei, Z., Chen, Z., He, Y. & Du, J. Thermal behavior in single track during selective laser melting of AlSi10Mg powder. *Appl. Phys. A Mater. Sci. Process.* **123**, 1–13 (2017).
2. Kobatake, H., Brillo, J., Schmitz, J. & Pichon, P. Y. Surface tension of binary Al-Si liquid alloys. *J. Mater. Sci.* **50**, 3351–3360 (2015).
3. Poletto, M. & Joseph, D. D. Effective density and viscosity of a suspension. *J. Rheol. (N. Y. N. Y).* **39**, 323–343 (2002).
4. Kou, S., Limmaneevichitr, C. & Wei, P. S. Oscillatory Marangoni flow: A fundamental study by conduction-mode laser spot welding. *Weld. J.* **90**, 229 (2011).
5. Loth, E. Drag of non-spherical solid particles of regular and irregular shape. *Powder Technol.* **182**, 342–353 (2008).
