# Supplementary material for: Pore elimination mechanisms during 3D printing of metals
Source: Nat Commun. 2019 Jul 12;10:3088. doi: 10.1038/s41467-019-10973-9 (PMC6625989; doi:10.1038/s41467-019-10973-9)
Supplement: Supplementary file 7 — Description of Additional Supplementary Files [file 41467_2019_10973_MOESM7_ESM.docx]

**Description of Additional Supplementary Files**

File Name: Supplementary Movie 1.

Description: Pore dynamics within the melt pool during LPBF processing of AlSi10Mg at laser power of 360 W and scan speed of 1 m/s.

File Name: Supplementary Movie 2.

Description: Pore dynamics within the melt pool during laser melting of an AlSi10Mg bare substrate at laser power of 360 W and scan speed of 1 m/s.

File Name: Supplementary Movie 3.

Description: Multi-physics modelling showing the dynamics of melt pool and temperature range in the melt pool during LPBF processing of AlSi10Mg at laser power of 360 W and scan speed of 1 m/s.

File Name: Supplementary Movie 4.

Description: Eliminating pores in feedstock powders using thermocapillary force during LPBF processing of Ti6Al4V at laser power of 210 W and scan speed of 0.6 m/s.

File Name: Supplementary Movie 5.

Description: Eliminating pores in the previously built layer using thermocapillary force during laser melting of Ti6Al4V at laser power of 210 W and scan speed of 0.6 m/s.
